# Applications of Repetitive Transcranial Magnetic Stimulation to Improve Upper Limb Motor Performance After Stroke: A Systematic Review

**DOI:** 10.1177/15459683231209722

**Published:** 2023-11-10

**Authors:** Afifa Safdar, Marie-Claire Smith, Winston D. Byblow, Cathy M. Stinear

**Affiliations:** 1 Department of Medicine, University of Auckland, Auckland, New Zealand; 2 Department of Exercise Sciences, University of Auckland, Auckland, New Zealand

**Keywords:** stroke, noninvasive brain stimulation, transcranial magnetic stimulation, theta burst stimulation, motor evoked potentials

## Abstract

**Background:**

Noninvasive brain stimulation (NIBS) is a promising technique for improving upper limb motor performance post-stroke. Its application has been guided by the interhemispheric competition model and typically involves suppression of contralesional motor cortex. However, the bimodal balance recovery model prompts a more tailored application of NIBS based on ipsilesional corticomotor function.

**Objective:**

To review and assess the application of repetitive transcranial magnetic stimulation (rTMS) protocols that aimed to improve upper limb motor performance after stroke.

**Methods:**

A PubMed search was conducted for studies published between 1st January 2005 and 1st November 2022 using rTMS to improve upper limb motor performance of human adults after stroke. Studies were grouped according to whether facilitatory or suppressive rTMS was applied to the contralesional hemisphere.

**Results:**

Of the 492 studies identified, 70 were included in this review. Only 2 studies did not conform to the interhemispheric competition model, and facilitated the contralesional hemisphere. Only 21 out of 70 (30%) studies reported motor evoked potential (MEP) status as a biomarker of ipsilesional corticomotor function. Around half of the studies (37/70, 53%) checked whether rTMS had the expected effect by measuring corticomotor excitability (CME) after application.

**Conclusion:**

The interhemispheric competition model dominates the application of rTMS post-stroke. The majority of recent and current studies do not consider bimodal balance recovery model for the application of rTMS. Evaluating CME after the application rTMS could confirm that the intervention had the intended neurophysiological effect. Future studies could select patients and apply rTMS protocols based on ipsilesional MEP status.

## Introduction

Stroke is the second leading cause of death and disability, and every year more than 12 million new cases are reported worldwide.^1^ Between 1990 and 2017 there was a 2-fold increase in the absolute number of people experiencing a stroke, dying from stroke, and surviving stroke with disability.^2^ Among those who survive a stroke, around 3-quarters experience impairment of the upper limb^3^ which affects functional activities and quality of life.^4^

Noninvasive brain stimulation (NIBS) techniques are experimental interventions that can be used to modulate cortical excitability and improve motor function after stroke. Neuromodulation is aimed at enhancing adaptive or suppressing maladaptive post-stroke neural re-organization.^5^ NIBS protocols can be classified as facilitatory or suppressive depending upon their effects on cortical excitability.^6^

The interhemispheric competition model influences the design of NIBS protocols. This model suggests that in a healthy brain, both hemispheres mutually and equally inhibit each other via transcallosal pathways. When stroke damages 1 hemisphere this balanced inhibition is disrupted because the ipsilesional hemisphere is unable to counteract the inhibitory effects of the contralesional hemisphere. Thus, this model assumes that after stroke there is reduced interhemispheric inhibition (IHI) acting on the contralesional hemisphere, whereas the ipsilesional hemisphere is not only damaged but excessively inhibited by the contralesional hemisphere.^7,8^

The interhemispheric competition model predicts that suppressing excitability of the contralesional hemisphere will reduce its IHI of the ipsilesional hemisphere. This is expected to indirectly produce an increase in ipsilesional excitability. However, a meta-analysis of 112 studies reported that there was no asymmetry in IHI, and no evidence that the contralesional hemisphere is hyperexcitable, 3 to 6 months post-stroke.^9^ This finding was subsequently supported by Xu et al^10^ who investigated premovement IHI in stroke patients during their first year of recovery. Premovement IHI was normal during the acute and subacute stages and only became abnormal at the chronic stage. This imbalance did not correlate with motor performance, which improved over time as premovement IHI became asymmetrical. Therefore, the authors concluded that asymmetric IHI is a consequence, rather than a cause, of poor motor recovery.^10^

The physiological function of IHI is not completely understood. Carson^11^ has advanced the idea that IHI may have a similar function to lateral inhibition mechanisms observed elsewhere in the cortex, and shape converging inputs to pyramidal neurons to increase the fidelity of their output. Transcallosal connections may have a primarily integrative function that fine-tunes descending motor output, rather than producing undifferentiated suppression of 1 hemisphere by the other.^11^

There is growing interest in individualizing the application of NIBS rather than applying it uniformly according to the interhemispheric competition model. According to the more recent bimodal balance recovery model,^12^ structural reserve of neural pathways determines functional recovery in stroke survivors. If the ipsilesional corticospinal tract (CST) can no longer transmit descending motor output from the cortex, the contralesional hemisphere may take on a compensatory role. This compensation may remain incomplete, and patients typically continue to experience severe upper limb impairment. Applying repetitive transcranial magnetic stimulation (rTMS) according to the interhemispheric competition model would involve suppressing contralesional excitability, which could undermine its compensatory function for patients with more ipsilesional damage. This is supported by the finding that suppressive protocols applied to the contralesional hemisphere can be detrimental for severely impaired patients.^13-18^

According to the bimodal model, some form of structural or functional test is needed to evaluate structural reserve in each patient to personalize their NIBS protocol.^12^ The motor evoked potential (MEP) status biomarker obtained with transcranial magnetic stimulation (TMS) can identify whether a patient has a functional ipsilesional CST, and direct the selection of NIBS protocol according to the bimodal balance model.

rTMS is a commonly used type of NIBS. High-frequency rTMS (≥5 Hz) can increase the excitability of neurons in primary motor cortex (M1), while stimulation frequencies below 5 Hz (low-frequency rTMS) can suppress excitability.^6^ In addition to simple high and low-frequency stimulation, rTMS can also be delivered in patterned protocols such as theta burst stimulation (TBS). Corticomotor excitability (CME) is typically facilitated by intermittent TBS (iTBS) and suppressed by continuous TBS (cTBS).^6^ There is interindividual variability in the response to rTMS protocols. The expected facilitation or suppression of excitability is typically observed in averaged data from groups of participants. Excitability can be unaffected, or modulated in the opposite direction, for some individuals.^19^ The purpose of this review is not to evaluate the efficacy of rTMS, but to understand the pattern of its application with respect to the interhemispheric competition model.

This systematic review aimed to identify, organize, evaluate, and summarize studies that investigated the effects of rTMS on upper limb impairment and function after stroke in the adult population. We were particularly interested in evaluating whether rTMS is applied to the contralesional hemisphere according to the interhemispheric competition model or the more recent bimodal balance model. The review is focused on the pattern of rTMS application, rather than its efficacy, and so a meta-analysis of the effects of rTMS was not carried out. The results of this review are expected to identify gaps in the current evidence base that could be addressed by tailored application of rTMS in future studies.

## Methods

### Literature Search

This is a systematic review of studies that applied rTMS to study upper limb impairment and function after stroke. PubMed was searched for the term “((((stroke) AND (contralesional OR unaffected OR non-lesioned)) AND (upper limb OR upper extremity OR hand OR arm)) AND (impairment OR function)) AND (MRI OR TMS OR transcranial magnetic stimulation)” to locate articles published from 1st January 2005 up to the 1st of November 2022. This search strategy identified 492 articles.

### Selection of Studies

**Figure 1.**
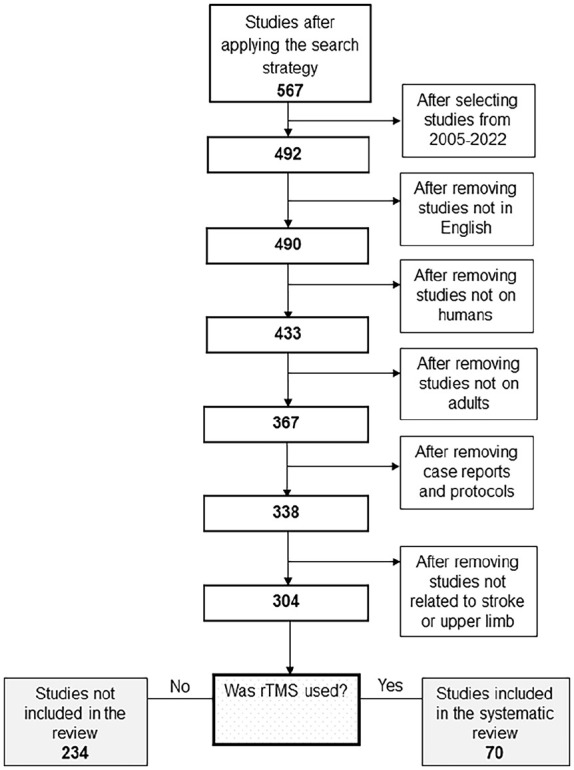
Of the 492 studies published from January 2005 to November 2022, 70 studies were included in this review that applied rTMS over the contralesional hemisphere to improve upper limb function after stroke.

The 492 studies identified were reviewed and excluded if they were not published in the English language, did not include human participants, were case reports or protocols, included a pediatric sample, were not related to stroke, or did not involve the upper limb. The remaining 304 studies were then reviewed to identify those that used rTMS and reported its effects on motor function or impairment of the upper limb post-stroke. The final set of 70 studies were included in this systematic review after satisfying the criteria shown in Figure 1.

### Data Extraction

Table 1 summarizes the data extracted from each study. Characteristics of interest included sample size, stage of post-stroke recovery, and whether the presence or absence of MEPs in the paretic upper limb (MEP status) was determined and/or used to select patients for the study. MEP status is a biomarker that reflects the functional integrity of the CST. Patients in whom MEPs can be elicited in the paretic upper limb are MEP+, and have better motor recovery and outcomes than those who are MEP−.^20^ The response to upper limb therapy also depends on MEP status at the chronic stage.^20^ For these reasons, MEP status is considered an important neurophysiological biomarker.^21^ Additional characteristics included whether CME was measured to see if the rTMS protocol had the intended neurophysiological effect, whether sham stimulation was used and the number of rTMS sessions.

**Table 1.** Data Extracted From Each Study.

Participant characteristics	• The stage of post-stroke recovery (Sub-acute: 0-6 mo, Chronic: >6 mo)
Study Characteristics	• Sample size • Number of sessions (single, multiple) • Primary outcome • Controls (sham intervention, healthy control group) • Corticomotor excitability measured (Yes, No)
NIBS characteristics	• Contralesional hemisphere stimulation (alone or combined with ipsilesional hemisphere facilitation) • Type (rTMS or TBS) • Protocol (facilitatory, suppressive) • Number of sessions (single, multiple)

## Categorization of studies

**Figure 2.**
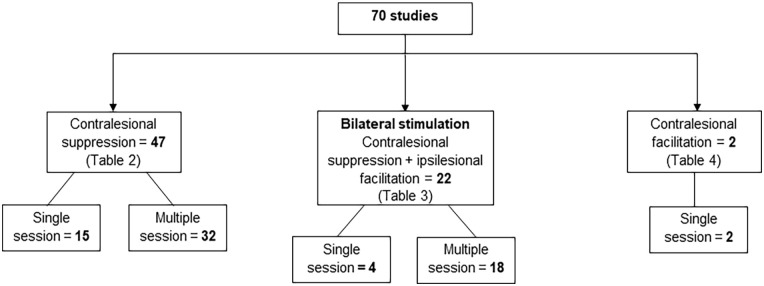
Distribution of studies according to the pattern of application of rTMS. One study suppressed the contralesional hemisphere in 1 session and facilitated it in another session, so it is included in both categories of contralesional suppression and contralesional facilitation.

The studies were divided into 3 categories. The first category included studies that only suppressed the contralesional hemisphere. The second category included studies that suppressed the contralesional hemisphere and facilitated the ipsilesional hemisphere, either sequentially or contemporaneously. The last category included studies that only facilitated the contralesional hemisphere. These studies were further categorized into single session or multiple session studies. Single session studies evaluated the immediate effects of rTMS, while multiple session studies evaluated potential cumulative effects (Figure 2).

## Search for Current Studies

The clinicaltrials.gov website was searched on 15th of December, 2022 to understand what protocols will be reported in future studies of rTMS interventions for upper limb rehabilitation in the adult stroke population. The website was searched by selecting “stroke” as the condition or disease, “transcranial magnetic AND motor” were entered for the other terms, and “recruiting, not yet recruiting, enrolling by invitation and active, not recruiting” options were selected for the recruitment status. This search strategy generated 91 results.

## Results

**Figure 3.**
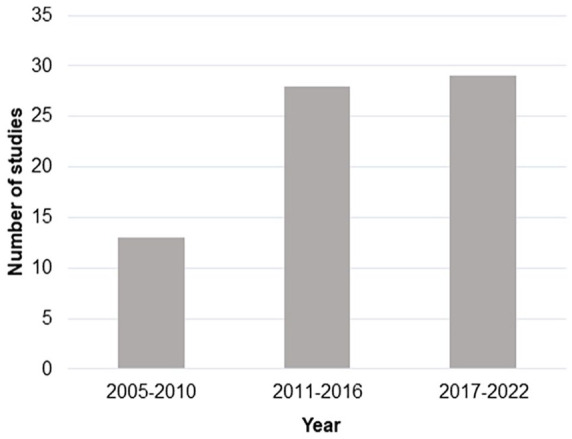
Number of studies published from January 2005 to November 2022 that applied rTMS over the contralesional hemisphere to improve upper limb function after stroke.

**Figure 4.**
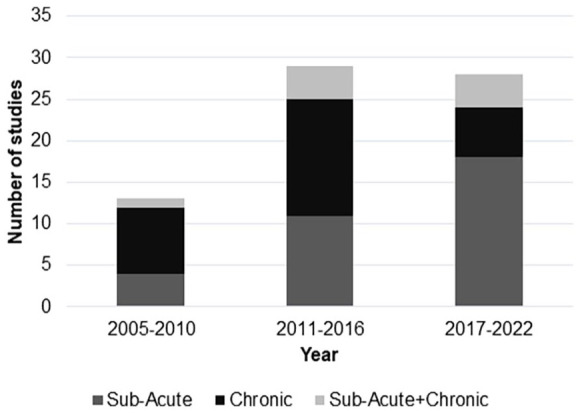
Number of studies published from January 2005 to November 2022 grouped according to time after stroke that applied rTMS over the contralesional hemisphere to improve upper limb function.

There were 70 studies that met the inclusion criteria (Figure 1) and most (57/70, 81%) were published after 2010 (Figure 3). Almost half of the studies (34/70, 49%) included 20 or fewer patients, and the majority were conducted with patients recruited only at the chronic stage (30/70, 43%) or at both the chronic and sub-acute stage (8/70, 11%; Figure 4).

Most studies (69/70, 98%) were designed to either suppress the contralesional hemisphere alone (47/70, 67%), or in combination with facilitation of the ipsilesional hemisphere (22/70, 31%). Only 2 studies applied rTMS over the contralesional hemisphere to facilitate it. The study by Sankarasubramanian et al^22^ suppressed the contralesional hemisphere in 1 session and facilitated it in another session.

Of the 47 studies that suppressed the contralesional hemisphere alone, 15 were single session studies and 32 were multiple session studies. There were 22 studies that suppressed the contralesional hemisphere in combination with ipsilesional facilitation, 4 were single session and 18 were multiple session studies. The 2 studies that facilitated the contralesional hemisphere applied rTMS in a single session.

Overall, 21/70 (30%) studies reported MEP status as a biomarker of ipsilesional corticomotor function. Around half of these studies (11/21) only included patients who were MEP+. Five of these 11 studies suppressed contralesional excitability to indirectly facilitate ipsilesional excitability, as per the interhemispheric competition model.^23-27^

Five studies that only included MEP+ patients directly facilitated ipsilesional excitability in combination with suppression of contralesional excitability.^28-32^ The remaining study that included only MEP+ participants applied facilitatory rTMS to the contralesional dorsal premotor cortex (cPMd).^15^

**Figure 5.**
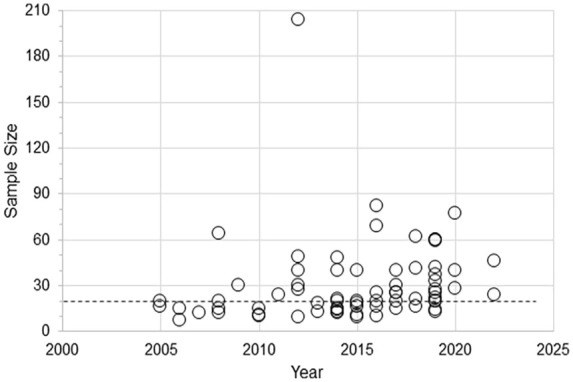
Scatter plot of sample size for studies published from January 2005 to November 2022 that applied rTMS over the contralesional hemisphere to improve upper limb function after stroke. *Note*. The dashed horizontal line represents the median sample size, n = 20.

More than half of the studies (37/70, 53%) measured corticomotor excitability to determine if the rTMS protocol had the intended neurophysiological effect. The remaining 33 studies only evaluated corticomotor function at baseline to determine resting or active motor threshold for the application of rTMS, but did not measure CME after the application of rTMS. Only 13/70 (19%) studies identified a primary outcome. The median sample size was 20 (range 7-204; Figure 5).

### Contralesional Suppression: Single Session Studies

Fifteen studies targeted the contralesional hemisphere with a protocol designed to suppress its excitability in a single session (Table 2). Three of these studies evaluated and reported MEP status. Cassidy et al and Takeuchi et al only included MEP+ patients, while Sankarasubramanian et al included both MEP+ and MEP− patients. Cassidy et al^23^ reported no facilitation of ipsilesional M1 excitability after suppressive contralesional rTMS in MEP+ patients. In contrast, Takeuchi et al^24^ and Sankarasubramanian et al^22^ reported that application of contralesional suppressive rTMS in MEP+ patients with mild upper limb impairment was associated with an increase in ipsilesional CME. The remaining 12 studies did not report MEP status and 5 of these 12 studies did not measure cortical excitability of the ipsilesional hemisphere after the application of rTMS.^33-37^

**Table 2.** Studies That Suppressed the Contralesional Hemisphere.

Ref #	Study	N	Stage	NIBS	Sessions	MEP status reported	MEP status criteria	CME measured	Primary outcome measure	Sham NIBS
^22^	Sankarasubramanian et al	15	C	rTMS	Single	Y	+ and −	Y		Y
^23^	Cassidy et al	11	C	rTMS	Single	Y	+	Y	Cortical excitability difference	Y
^24^	Takeuchi et al	20	C	rTMS	Single	Y	+	Y		Y
^25^	Noh et al	22	SA	rTMS	Multiple	Y	+	Y		N
^26^	Lüdemann-Podubecká et al	40	SA	rTMS	Multiple	Y	+	Y		Y
^27^	Carey et al	12	C	rTMS	Multiple	Y	+	N	TEMPA	N
^33^	Liepert et al	12	SA	rTMS	Single	N		N		Y
^34^	Nowak et al	15	SA	rTMS	Single	N		N		Y
^35^	Kondo et al	13	B	rTMS	Single	N		N		N
^36^	Grefkes et al	11	SA	rTMS	Single	N		N		Y
^37^	Dafotakis et al	12	SA	rTMS	Single	N		N		Y
^38^	Bonin Pinto et al	27	B	rTMS	Multiple	Y	+ and −	Y	JHFT and UE-FM	Y
^39^	Neva et al	37	C	TBS	Multiple	Y	+ and −	Y	STT and WMFT	Y
^40^	Luk et al	24	SA	rTMS	Multiple	Y	+ and −	Y		Y
^41^	Takeuchi et al	20	C	rTMS	Single	N		Y		Y
^42^	Theilig et al	24	B	rTMS	Multiple	N		Y		Y
^43^	Rose et al	21	C	rTMS	Multiple	N		Y	WMFT	Y
^44^	Galvão et al	20	C	rTMS	Multiple	N		N	MAS	Y
^45^	Seniów et al	40	SA	rTMS	Multiple	N		N		Y
^46^	Bashir et al	16	C	rTMS	Single	N		Y		N
^47^	Mansur et al	16	B	rTMS	Multiple	N		N		Y
^48^	Mello et al	18	SA	rTMS	Multiple	N		Y		Y
^49^	Pan et al	42	B	rTMS	Multiple	N		N	WMFT	N
^50^	Tamashiro et al	59	B	rTMS	Multiple	N		N		N
^51^	Tosun et al	25	SA	rTMS	Multiple	N		N	UE-FM, BRSs, and UE-MI	N
^52^	Conforto et al	30	SA	rTMS	Multiple	N		N	Feasibility and safety	Y
^53^	Kim et al	82	SA	rTMS	Multiple	N		N	K-MBI	N
^54^	Blesneag et al	16	SA	rTMS	Multiple	N		Y		Y
^55^	Kim et al	77	SA	rTMS	Multiple	N		N	BBT	N
^56^	Tretriluxana et al	16	SA	rTMS	Single	N		Y		Y
^57^	Lüdemann-Podubecká et al	10	SA	rTMS	Single	N		Y		Y
^58^	Tretriluxana et al	9	C	rTMS	Single	N		Y		Y
^59^	Vongvaivanichakul et al	14	C	rTMS	Single	N		Y		Y
^60^	Aşkın et al	40	C	rTMS	Multiple	N		N		N
^61^	Etoh et al	20	C	rTMS	Multiple	N		N		Y
^62^	Dos Santos et al	20	C	rTMS	Multiple	N		Y		Y
^63^	Ueda et al	25	C	rTMS	Multiple	N		N		N
^64^	Niimi et al	13	C	rTMS	Multiple	N		N		N
^65^	Hara et al	25	C	rTMS	Multiple	N		N		N
^66^	Matsuura et al	20	SA	rTMS	Multiple	N		N		Y
^67^	Kakuda et al	15	C	rTMS	Multiple	N		Y		N
^68^	Fregni et al	15	C	rTMS	Multiple	N		Y		Y
^69^	Etoh et al	18	C	rTMS	Multiple	N		N		Y
^70^	Chen et al	40	SA	rTMS	Multiple	N		N		Y
^71^	Koyama et al	15	C	rTMS	Multiple	N		N		N
^72^	Kakuda et al	204	C	rTMS	Multiple	N		N		N
^73^	Tretriluxana et al	9	C	rTMS	Single	N		Y		Y

Abbreviations: B, Both; BBT, Box and Block test; BRS, Brunnstrom recovery scale; C, Chronic; CME, Corticomotor excitability; GS, Grip Strength; JHFT, Jebsen-Taylor hand function test; K-MBI, Korean version of Modified Barthel Index; MAS, Modified Ashworth scale; MEP, Motor evoked potentials; SA, Sub-acute; STT, serial targeting task; TEMPA, Test Evaluant la performance des Membres sup’erieurs des Personnes Aˆ ge’es; WMFT, Wolf Motor Function Test; UE-MI, Upper Extremity Motricity Index.

### Contralesional Suppression: Multiple Session Studies

The largest group of studies suppressed the contralesional hemisphere in multiple sessions using either rTMS or cTBS (Table 2). Of these 32 studies, 6 evaluated and reported MEP status. Noh et al,^25^ Lüdemann-Podubecká et al,^26^ and Carey et al^27^ only recruited patients who were MEP+ and applied suppressive contralesional rTMS. Bonin Pinto et al,^38^ Neva et al,^39^ and Luk et al^40^ recruited both MEP+ and MEP− patients.

Out of the remaining 32 studies, only 12 studies measured the ipsilesional CME to confirm if the suppression of the contralesional hemisphere had the intended neurophysiological effect.

### Bilateral Stimulation: Single Session Studies

Out of 70 studies included in this review, 4 facilitated the ipsilesional hemisphere and suppressed the contralesional hemisphere in a single session (Table 3). Two studies separately facilitated the ipsilesional hemisphere and suppressed the contralesional hemisphere in consecutive sessions in both studies, iTBS was used to facilitate the ipsilesional hemisphere in 1 session, and cTBS to suppress the contralesional hemisphere in another session, with sham stimulation delivered in a third session.^28,74^ MEP status was evaluated and reported in both studies. One study included both MEP+ and MEP− patients,^74^ while the other recruited only MEP+ patients.^28^ An increase in ipsilesional excitability was reported when iTBS was applied to the contralesional M1^28^ and a reduction in ipsilesional excitability was reported when cTBS was applied to the contralesional M1.^74^

**Table 3.** Studies That Suppressed the Contralesional Hemisphere and Facilitated the Ipsilesional Hemisphere.

Ref #	Study	N	Stage	NIBS	Sessions	MEP status reported	MEP status criteria	CME measured	Primary outcome measure	Sham NIBS
^28^	Ackerley et al	13	C	TBS	Single	Y	+	Y		Y
^29^	Kwon et al	20	B	rTMS+TDCS	Multiple	Y	+	Y		Y
^30^	Du et al	60	SA	rTMS	Multiple	Y	+	Y	UE-FM	Y
^31^	Takeuchi et al	30	C	rTMS	Multiple	Y	+	Y		Y
^32^	Khan and Chevidikunnan	20	SA	TBS	Single	Y	+	Y	NHPT	N
^74^	Ackerley et al.	10	C	TBS	Single	Y	+ and −	Y		Y
^75^	Takeuchi et al	27	C	rTMS+TDCS	Single	N		Y		Y
^76^	Cho et al	30	SA	rTMS	Multiple	Y	+ and −	N	UE-FM	N
^77^	Fleming et al	25	B	rTMS	Multiple	Y	+ and −	Y		Y
^78^	Meng et al	28	SA	rTMS	Multiple	Y	+ and −	Y		Y
^79^	Watanabe et al	21	SA	rTMS	Multiple	N		Y		Y
^80^	Kim et al	40	SA	rTMS	Multiple	N		N		N
^81^	Du et al	69	SA	rTMS	Multiple	Y	+ and −	Y	UE-FM	Y
^82^	Du et al	46	SA	rTMS	Multiple	Y	+ and −	Y		Y
^83^	Nicolo et al	41	SA	TBS+TDCS	Multiple	N		N	Δ of slope of improvement during recovery and during treatment	Y
^84^	Talelli et al	49	C	TBS	Multiple	N		N	NHPT, JHFT, and maximal grasp and pinch grip dynamometry	Y
^85^	Málly et al	64	C	rTMS	Multiple	N		N		N
^86^	Wang et al	48	SA	rTMS	Multiple	N		Y		Y
^87^	Chouinard et al	7	C	rTMS	Multiple	N		N		N
^88^	Khan et al	60	SA	TBS	Multiple	N		Y	UE-FM	N
^89^	Long et al	62	SA	rTMS	Multiple	N		N		Y
^90^	Lee et al	33	SA	rTMS	Multiple	N		N		N

Abbreviations: B, Both; C, Chronic; CME, Corticomotor excitability; JHFT, Jebsen-Taylor hand function test; MEP, Motor evoked potentials; NHPT, Nine Hole Peg test; SA, Sub-acute.

Two studies applied NIBS to both hemispheres contemporaneously in a single session. Takeuchi et al^75^ applied suppressive rTMS to the contralesional hemisphere and anodal transcranial direct current stimulation (TDCS) to the ipsilesional hemisphere at the same time. MEP status was not reported, and an increase in ipsilesional excitability was observed. Khan and Chevidikunnan^32^ only recruited MEP+ patients and applied iTBS to the ipsilesional hemisphere and cTBS to the contralesional hemisphere in a single session. They reported an increase in ipsilesional excitability and a reduction in contralesional excitability after the application of TBS.^32^

### Bilateral Stimulation: Multiple Session Studies

Out of 70 studies included in this review, 18 facilitated the ipsilesional hemisphere and suppressed the contralesional hemisphere in multiple sessions (Table 3). About 14 out of 18 studies applied rTMS to both hemispheres at the same time in multiple sessions. All of these studies applied rTMS to suppress contralesional excitability and facilitate ipsilesional excitability. Six of these 14 studies evaluated and reported patients’ MEP status. Three studies recruited only MEP+ patients,^29,30,41^ and 3 studies included both MEP+ and MEP− patients.^76-78^

Four out of 18 applied suppressive stimulation to 1 group of patients and facilitatory stimulation to another group of stroke patients, and both were compared to a third group who had sham stimulation.^79-82^ Two of the 4 studies evaluated the MEP status of the stroke patients and included both MEP+ and MEP− patients.^81,82^

### Contralesional Facilitation: Single Session Studies

Two studies facilitated the contralesional hemisphere in a single session (Table 4). Both studies evaluated and reported MEP status. Liao et al^15^ only included MEP+ patients, while Sankarasubramanian et al^22^ included both MEP+ and MEP− patients. Liao et al reported that patients with severe baseline upper limb impairment, and less IHI from contralesional to ipsilesional hemisphere, improved bimanual force and neuromuscular coordination and ipsilesional excitability after cPMd was facilitated with 5 Hz rTMS. On the other hand, patients with mild upper limb impairment experienced improvement in bimanual force and ipsilesional excitability only after ipsilesional M1 was facilitated with 5 Hz rTMS.^15^ Sankarasubramanian et al^22^ reported that facilitation of the contralesional hemisphere improved clinical outcome measures in patients with greater clinical impairment but did not increase ipsilesional excitability.

**Table 4.** Studies That Facilitated the Contralesional Hemisphere.

Ref #	Study	N	Stage	NIBS	Sessions	MEP status reported	MEP status criteria	CME measured	Sham NIBS
^15^	Liao et al	14	SA	rTMS	Single	Y	+	Y	N
^22^	Sankarasubramanian et al	15	C	rTMS	Single	Y	+ and −	Y	Y

Abbreviations: SA, Sub-acute; C, Chronic; B, Both; MEP, Motor evoked potential; CME, Corticomotor excitability.

### Current Studies

**Figure 6.**
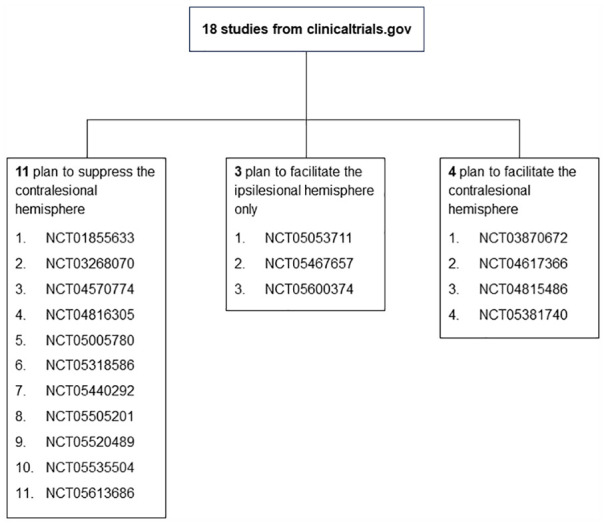
Distribution of current studies that plan to apply rTMS to improve hand function after stroke. The numbers in each box are clinical trial registration numbers from clinicaltrials.gov website.

Out of 91 studies retrieved from clinicaltrials.gov, there were 27, 18 studies with a clearly defined protocol for applying rTMS to influence upper limb motor outcomes in adult stroke patients (Figure 6). Eleven of these 18 studies plan to suppress the contralesional hemisphere using either low frequency rTMS (LF-rTMS) or cTBS alone or combined with ipsilesional facilitation. Three of these 18 studies plan to directly facilitate the ipsilesional hemisphere with rTMS. The remaining 4 studies plan to facilitate the contralesional hemisphere. Two out of these 4 studies are from the same authors of the study included in this review that also applied facilitatory rTMS to the contralesional hemisphere.^22^ Five of the 18 studies do not specify inclusion or exclusion criteria related to patients’ level of upper limb impairment. Only 2 studies plan to use MEP status as a biomarker for the application of rTMS and they plan to recruit only MEP+ patients.

## Discussion

This review found that the interhemispheric competition model continues to strongly influence the application of rTMS in studies designed to improve upper limb motor performance after stroke. Most studies were designed to either suppress the contralesional hemisphere alone (47/70, 67%), or in combination with facilitation of the ipsilesional hemisphere (22/70, 31%). However, evidence for the interhemispheric competition model may not be as compelling as first thought.

A large meta-analysis found no asymmetry in IHI at the sub-acute or chronic stages of stroke.^9^ Moreover, fMRI studies show that contralesional motor areas, particularly the cPMd, support residual motor function by exerting facilitatory influences on the surviving sensorimotor cortex of the ipsilesional hemisphere.^16,74^ It therefore may be unhelpful to take a 1-size-fits-all approach and suppress activity in the contralesional hemisphere for all patients.

The bimodal balance recovery model can be used to guide individualized application of rTMS protocols. This model predicts that mild to moderately impaired patients with adequate structural reserve will benefit from facilitation of the ipsilesional hemisphere. Whereas severely impaired patients with little structural reserve rely on the contralesional hemisphere and may benefit from its facilitation.^12^ There is some support for these predictions,^13-16,18,22^ however this review found that most studies do not evaluate corticospinal reserve to personalize the application of NIBS. The use of biomarkers to evaluate CST function might be beneficial for clinical practice, and time effective for patients, families, and clinicians.^91^

MEP status is a biomarker that could be used to select rTMS protocols for individual patients. Five studies applied facilitatory rTMS to the ipsilesional M1 together with suppressive rTMS to the contralesional M1 in MEP+ patients. All confirmed that ipsilesional CME was facilitated by the rTMS intervention, and all reported a positive result in improving upper limb function. In contrast, the 5 studies that only suppressed contralesional excitability in MEP+ patients produced equivocal results. Four of the 5 studies measured ipsilesional CME before and after the intervention as a manipulation check. However, only 2 studies of mild to moderately impaired patients reported an increase in ipsilesional CME after suppressive rTMS of the contralesional hemisphere.^24,25^ This aligns with the results of study^13^ of patients with severe upper limb impairment which found that suppressive TDCS of the contralesional hemisphere had no effect on ipsilesional CME and worsened paretic upper limb motor function. Similarly, a multisite trial applied suppressive rTMS to the contralesional hemisphere in 167 patients 3 to 12 months post-stroke for a period of 6 weeks and reported no differences in motor function between the active rTMS and sham rTMS groups.^92^ These results indicate that suppressive NIBS interventions delivered to the contralesional M1 of stroke patients do not consistently facilitate ipsilesional CME or benefit paretic upper limb motor function.

Facilitating ipsilesional excitability is less likely to be beneficial for MEP− patients, who have severely limited descending CST output from the ipsilesional M1. Instead, facilitatory NIBS protocols could be applied to the contralesional hemisphere to enhance its compensatory role. Previous fMRI studies have shown that contralesional motor cortex activity, particularly in the cPMd,^16^ is associated with better upper limb motor recovery and function in severely impaired patients.^17^ Conversely, TMS virtual lesions of cPMd and M1 result in slower hand tapping speeds, and timing errors in sequential paretic finger movements, in severely impaired stroke patients.^18^ McCambridge et al reported that facilitatory anodal TDCS over the contralesional hemisphere increased ipsilesional CME and improved upper limb motor performance in severely impaired stroke patients. Interestingly, suppressive cathodal TDCS applied over the contralesional hemisphere in a separate session had no effect on the excitability of cM1 or motor performance.^14^ Facilitating the contralesional hemisphere could therefore be beneficial for MEP− patients.

This review identified only 2 studies that have facilitated the contralesional hemisphere to date using rTMS. Both reported beneficial effects of contralesional facilitation on paretic upper limb performance after a single session (Table 4). Sankarasubramanian et al selected both MEP+ and MEP− patients for their study and applied both the standard approach of contralesional suppression and the new approach of contralesional facilitation, in addition to sham stimulation. While the standard approach benefited mild to moderately impaired stroke patients, it was the facilitation of contralesional hemisphere that improved motor performance and enhanced excitability in severely impaired stroke patients.^22^ Similar findings were reported by Liao et al^15^ These studies provide preliminary evidence for the potential benefits of contralesional facilitation in MEP−, severely impaired patients. However, neither of these studies combined rTMS with therapy, sample sizes were small (14 and 15), and the potential cumulative effects of multiple sessions are currently unknown (Table 4).

The studies included in this review share some common limitations. Almost half of studies (33/70, 47%) did not measure CME after the application of rTMS. This means these studies did not confirm whether suppressing the contralesional hemisphere and/or facilitating the ipsilesional hemisphere had the expected effects on CME based on the interhemispheric competition model. The failure to carry out manipulation checks undermines confidence in the results, and may contribute to a lack of detectable effects of rTMS.

Sample sizes were typically small with a median of 20 participants (range 7-204). Just under half of the studies selected for this systematic review (30/70, 43%) recruited patients at the chronic stage. A further 11% of studies (8/70) included patients at both the sub-acute and chronic stages of stroke. While such an approach might increase participant numbers, including patients at a single stage of recovery can reduce the potentially confounding effects of a subset of participants experiencing spontaneous recovery. Further work is needed to explore the effects of rTMS on motor recovery at the subacute stages of stroke.

The reviewed studies also have various strengths. Most compared the effects of rTMS in stroke patients with sham stimulation or a healthy control group with appropriate blinding. Most studies applied the intervention in multiple sessions to study the cumulative effects of rTMS.

This systematic review has some potential limitations. Only 1 database was searched for the studies. This review only included rTMS and did not include other types of NIBS, such as TDCS. Moreover, this review did not include studies that facilitate the ipsilesional hemisphere alone, as the focus of the review was to identify how the interhemispheric competition has influenced the application of rTMS over the contralesional hemisphere. Future reviews of studies that facilitate the ipsilesional alone are warranted.

## Current Studies

The majority of current studies registered on clinicaltrials.gov are planning to apply rTMS according to the interhemispheric competition model. More than half (11/18, 61%) are designed to indirectly facilitate the ipsilesional hemisphere by suppressing the contralesional hemisphere. Five of these 18 studies (30%) do not specify the level of impairment of included patients. Applying suppressive rTMS to the contralesional hemisphere in severely impaired and possibly MEP− patients may be detrimental as the contralesional hemisphere may have an important compensatory role for these patients. On the other hand, only 3 out of 18 (17%) plan to promote upper limb function by directly facilitating the ipsilesional hemisphere, while a further 4/18 (22%) depart from the interhemispheric competition model by facilitating the contralesional hemisphere. This review indicates that the former approach is more likely to benefit MEP+ patients, while the latter approach is more likely to benefit MEP− patients. Unfortunately, only 2 out of 27 studies (7%) plan to use the MEP status biomarker to select participants. This will make it difficult to draw strong conclusions from these studies about how to select patients most likely to benefit from the tested rTMS interventions.

## Conclusion

Despite well-documented limitations, the interhemispheric competition model continues to strongly influence the design of rTMS trials that aim to improve upper limb motor function after stroke. Most recent studies do not apply rTMS according to the principles of the bimodal balance recovery model, nor have they selected patients based on the integrity of key descending pathways necessary for recovery of dexterous motor function. These factors potentially impede the advancement of neuromodulation approaches for upper limb motor function after stroke. Future studies could usefully select patients and rTMS protocols according to MEP status. Direct facilitation of the ipsilesional hemisphere might be more effective for MEP+ patients than attempting to indirectly facilitate ipsilesional excitability by suppressing the contralesional hemisphere. Facilitation of the contralesional hemisphere might be appropriate for MEP− patients for whom the contralesional hemisphere may play an important compensatory role. Regardless of the target of rTMS, the evaluation of CME after its application could ascertain whether the intervention had the intended neurophysiological effect of increasing ipsilesional excitability. Prospective studies using MEP status to stratify patients in trials of rTMS are needed to test these ideas.
